# Hauora Māori – Māori health: a right to equal outcomes in primary care

**DOI:** 10.1186/s12939-023-02071-6

**Published:** 2024-02-27

**Authors:** Nicolette Sheridan, Rawiri McKree Jansen, Matire Harwood, Tom Love, Timothy Kenealy, The Primary Care Models Study Group, Nelson Aguirre-Duarte, Bruce Arroll, Carol Atmore, Jenny Carryer, Peter Crampton, Anthony Dowell, Tana Fishman, Robin Gauld, Karen Hoare, Gary Jackson, Ngaire Kerse, Debra Lampshire, Lynn McBain, Jayden MacRae, Jane Mills, John Øvretveit, Teuila Percival, Roshan Perera, Martin Roland, Debbie Ryan, Jacqueline Schmidt-Busby, Tim Stokes, Maria Stubbe

**Affiliations:** 1 Massey University, Auckland, Aotearoa New Zealand; 2 Te Aka Whai Ora – Māori Health Authority, Wellington, Aotearoa New Zealand; 3 University of Auckland, Auckland, Aotearoa New Zealand; 4 Sapere Research Group, Wellington, Aotearoa New Zealand

**Keywords:** Māori, Health equity, Models of care, Primary care, Nursing, Patient outcomes, Immunisations, Emergency department attendance, Avoidable hospitalisations, Ambulatory sensitive hospitalisations, Deprivation

## Abstract

**Background:**

For more than a century, Māori have experienced poorer health than non-Māori. In 2019 an independent Tribunal found the Government had breached Te Tiriti o Waitangi by “failing to design and administer the current primary health care system to actively address persistent Māori health inequities”. Many Māori (44%) have unmet needs for primary care. Seven models of primary care were identified by the funders and the research team, including Māori-owned practices. We hypothesised patient health outcomes for Māori would differ between models of care.

**Methods:**

Cross-sectional primary care data were analysed at 30 September 2018. National datasets were linked to general practices at patient level, to measure associations between practice characteristics and patient health outcomes. Primary outcomes: polypharmacy (≥ 55 years), HbA1c testing, child immunisations, ambulatory sensitive hospitalisations (0–14, 45–64 years) and emergency department attendances. Regressions include only Māori patients, across all models of care.

**Results:**

A total of 660,752 Māori patients were enrolled in 924 practices with 124,854 in 65 Māori-owned practices.

Māori practices had: no significant association with HbA1c testing, ambulatory sensitive hospitalisations or ED attendances, and a significant association with lower polypharmacy (3.7% points) and lower childhood immunisations (13.4% points).

Māori practices had higher rates of cervical smear and cardiovascular risk assessment, lower rates of HbA1c tests, and more nurse (46%) and doctor (8%) time (FTE) with patients.

The average Māori practice had 52% Māori patients compared to 12% across all practices. Māori practices enrolled a higher percentage of children and young people, five times more patients in high deprivation areas, and patients with more multimorbidity.

More Māori patients lived rurally (21.5% vs 15%), with a greater distance to the nearest ED. Māori patients were more likely to be dispensed antibiotics or tramadol.

**Conclusions:**

Māori practices are an expression of autonomy in the face of enduring health system failure. Apart from lower immunisation rates, health outcomes were not different from other models of care, despite patients having higher health risk profiles. Across all models, primary care need was unmet for many Māori, despite increased clinical input. Funding must support under-resourced Māori practices and ensure accountability for the health outcomes of Māori patients in all models of general practice.

## Background

For more than a century, Māori have experienced poorer health than non-Māori despite the 1840 Treaty of Waitangi (Te Tiriti o Waitangi) guaranteeing Māori the same rights and privileges as British citizens living in Aotearoa New Zealand. Māori retained tino rangatiratanga (sovereignty) over their taonga (treasures); health is a taonga. Nevertheless, current life expectancy at birth is 7.1 years shorter for Māori than non-Māori, with Māori experiencing many of the worst health outcomes of any population in Aotearoa New Zealand [1].

As far back as 1934 the Māori death rate was more than double that of non-Māori. Mortality from tuberculosis, the most common cause of death amongst Māori, was about 10 times the rate for non-Māori and the death rate from typhoid fever was nearly 40 times [2]. In 1938, infant mortality was four times higher for Māori than non-Māori. Maternity care became free in 1939 and Māori infant mortality fell steadily. With vaccinations and other measures, typhoid outbreaks were rare by the 1950s and tuberculosis was no longer a significant cause of death among Māori by 1964 [3].

Pomare, in 1980, first reported that patterns of mortality were systematically higher for Māori than non-Māori across multiple common, amendable diseases [4]. Subsequent reports brought the analysis up to 2005. Difficulties in accurate counting of ethnicity data have historically led to underestimates of Māori mortality and morbidity [5, 6].

Recent data report Māori children are 40% more likely to experience an ambulatory sensitive hospitalisation (ASH) than non-Māori children, most commonly for asthma, respiratory infections, dental issues, gastroenteritis and cellulitis [7]. For adults aged 35 + years, the rate of cardiovascular disease hospitalisation per 100,000 people, in 2012–14, was 3186 for Māori, and 1939 for non-Māori [8]. The rate for stroke was 366 for Māori and 208 for non-Māori; the rate for heart failure was 546 for Māori and 136 for non-Māori. The rheumatic heart disease hospitalisation rate, for those aged 15 + years, was 39 for Māori and 8 for non-Māori. In 2020, an estimated 46,400 (10.5%) Māori adults had diabetes compared to 7.1% of non-Māori and were almost twice as likely to experience diabetes-related limb amputation or renal failure [7].

Reid argues that inequitable health outcomes are the result of “a reliance on singular ‘one size fits all’ policies and processes as a demonstration of ‘treating everyone equally’” [9]. Major health system reforms took effect from 1 July 2022, under the Pae Ora (Healthy Futures) Act. An independent statutory authority, Te Aka Whai Ora—Māori Health Authority, is leading improvement in Māori health, with mātauranga Māori (Māori knowledge) accepted as central to achieving health gains. Te Aka Whai Ora is not a separate health system for Māori, but an entity to co-design and co-commission for the new health system. A new single national entity, Te Whatu Ora—Health New Zealand, is responsible for commissioning and delivery of hospital and health services. Community localities, yet to be established, will connect general practices, Primary Health Organisations (PHOs) and other primary care entities.

### Aotearoa New Zealand

Aotearoa New Zealand is an island country in the southwestern Pacific Ocean. It constitutes two large islands—the North Island and the South Island, a smaller third island (Stewart Island) and many islets. The land has a total area of 267,710 km^2^ (103,363 mi^2^), about 10% larger than the UK, has a total coastline of 15,134 km (9,403.8 mi) making it the nineth longest in the world, and an exclusive economic zone that covers 4,083,744 km^2^ (1,576,742 mi^2^), approximately 15 times the land area of the country. The total population at June 2022 was estimated at about 5.1 million of which 17.4% were Māori [10, 11].

First discovered and inhabited by Māori tribes in about AD 800 to 1200 [12, 13], Aotearoa was the name given by Māori to this land. The other name, New Zealand, was given by a Dutch explorer recorded as ‘discovering’ this country, “some hundreds of years into Māori tenure” [9]. Māori are tangata whenua—people of the land—and comprise about forty iwi (tribes) and hapu (sub-tribes), who individually derive their identity from the mountains, rivers, and lakes [14]. The landscape is mountainous with dense forests, fjords and glaciers in the southwest, volcanoes and geothermal springs in the central North Island, and miles of coastal beaches.

### Colonisation

Aotearoa New Zealand was colonised under an international legal principle known today as the Doctrine of Discovery, justified on religious and racial ideas of European and Christian superiority [15]. It allowed British settlers to stake legal claims to the land and property rights of Māori. Under English law land was available for its Discovery claims, first, if no other European country was in actual possession when English explorers arrived, and second, even if it was occupied by Native people if it was legally “vacant” and “unused” or terra nullius. By the 1930s very little tribal land remained in Māori ownership (today it amounts to 5% of the total landmass of Aotearoa). The doctrine of Native Title was not fully reinstated into Aotearoa New Zealand common law until 2003 [14].

Colonisation is the “violent denial of the rights of Indigenous peoples to continue governing themselves in their own lands” [16]. Disease and warfare decimated the population and legislation criminalized the Māori way of life [14]. British settler colonisation excluded Māori from reclaiming the right of Indigenous peoples to govern themselves. The right to decolonise under the “blue water” doctrine was restricted to Indigenous peoples whose lands were governed by colonisers, separated by an ocean, residing in their home country. “Settlers saw the land as a better Britain in the Pacific, but they increasingly claimed a certain permanence – while turning away from the fact that in settling themselves they were continually unsettling us” [16].

### Te Tiriti o Waitangi

The Crown sought to negotiate a treaty to acquire Māori consent to establish a form of civil government. On 6 February 1840, Te Tiriti o Waitangi was signed at Waitangi in the Bay of Islands by Captain William Hobson before the Māori text of Te Tiriti was taken around Northland to obtain Māori signatures. Copies were also sent around the country for signing. By the end of that year, over 500 Māori had signed Te Tiriti. The English text was signed only at Waikato Heads and Manukau. There is dispute over the translation of two of the three articles [17, 18]. The English version captures the principles of the Doctrine of Discovery; the Māori version asserts a plan for a future bound more in respectful separation [19, 20].^1^

Aotearoa New Zealand has a unicameral Parliament which has no formal limits to its law-making power [21, 22]. Te Tiriti o Waitangi is not part of the domestic law but is legally relevant when incorporated into statute, a relatively recent phenomenon [14]. The existence of Te Tiriti o Waitangi, a single treaty of cession, and legal institutions including the Māori Land Court and the Waitangi Tribunal,^2^ are unique to Aotearoa New Zealand. The recent Tribunal inquiry into the health system found racism to be endemic [1]. Colonization, built on racism, takes on subtle forms—intolerance, prejudice, and discrimination; “Colonisation is a process of dispossession and control, not a historical artefact” [16].

### Māori provision of primary health care

Advances in Māori health were associated with Māori leadership and tino rangatiratanga, Māori self-determination, within a context of cultural, social, and economic development. Durie (1999) identified three periods of Māori leadership: 1900–1930 witnessed greater Māori participation in health by tribal leaders and recent Māori medical graduates, despite the Department of Health maintaining a central control which led to displaced community leaders, the Tohunga Suppression Act (1907), and little support for Māori nurses [23]; 1931–1975 involved Māori women, who, although regarded as essentially a support to health professionals, connected Māori to mainstream health services, established the Māori Women’s Health League (1937) and Māori Women’s Welfare League (1951), and entered the health professions, especially nursing; by 1976–1992 Māori health initiatives were active in most Māori communities and Māori models of health were making more explicit the implications of culture to health with due recognition of tikanga Māori.

Iwi gained experience in providing health programmes in the 1980s, with many employing Māori community health workers and nurses to undertake health screening (cervical cancer, hearing loss), provide health education (pregnancy, asthma, diabetes) or counselling (mental health) and advocacy for Māori consumers. Iwi were interested in health in the context of Māori development. Across Aotearoa New Zealand Māori primary health care organisations continued to emerge, enhancing a Māori identity. Some came to provide general practice services, including clinics on marae.

Recently, the Waitangi Tribunal concluded government had committed a serious breach of Te Tiriti: “Māori primary health organisations were underfunded from the outset. We further found that ongoing resourcing was a significant issue too: the funding arrangements for the primary health care system disadvantage primary health organisations and providers that predominately serve high-needs populations, particularly Māori primary health organisations and providers. The Crown has been aware of these failures for well over a decade but has failed to adequately amend or replace the current funding arrangements” [1].

The Waitangi Tribunal found the Government had “breached the Treaty of Waitangi by failing to design and administer the current primary health care system to actively address persistent Māori health inequities” [1]. In 2020, 43.6% of Māori reported unmet need for primary care, most commonly due to cost [7]. The current system of delivering primary care across the country has, for more than 80 years, been largely based on general practices operating within a business model that is a legacy of colonisation and imported capitalism. We are not aware of any previous national study that examined variation in health outcomes, for Māori, at the level of practice model of care.

### This study

The aim of this study was to determine whether health outcomes for Māori varied across models of primary care. This paper reports results on Māori practices and Māori patients within a broader national study that examined associations between models of primary care and health outcomes across the whole population [24]. Māori, compared to non-Māori, had poorer health outcomes independent of model of care in which they were enrolled.

The current paper describes a history and context for Māori practices and Māori patients that was not possible in a more general paper. Further, by including only Māori patients in regression models we could address the hypothesis that health outcomes for Māori patients would differ between models of care. This was examined by multi-level modelling of patient outcomes with model of care as an explanatory variable, adjusted for practice and patient factors. Equity, by model of care, was assumed if models showed no significant association with patient outcomes.

## Methods

A cross sectional, observational study was conducted of all Aotearoa New Zealand general practices and enrolled patients as of 30 September 2018. Māori investigators provided governance to the project with respect to selecting outcomes and explanatory variables and interpreting results. What follows is a brief description of methods; further details can be found in the primary outcomes report and supplementary files [24]. The methods used in the two studies are the same but the populations differ; the regressions reported here included Māori patient data only.

### Data sources

Data came from national datasets, held by the Ministry of Health, and from practice information held by PHOs. National datasets included PHO registers, inpatient, outpatient, laboratories, pharmaceutical dispensing, immunisations, the Virtual Diabetes Register (VDR) [25], NZDep2018 Index of Deprivation [26], the Index of Multiple Deprivation (IMD) [27] and the Measuring Multimorbidity Index (M3) [28], all available at patient level. A patient unique identifier, their National Health Index (NHI), is used throughout the health system.

Ten PHOs, with 292 practices, contributed patient-level data. Data from appointment books was used to calculate number and length of consultations and the profession of the clinician seen, for face-to-face consultations, but not telephone, email or other contacts.

Three measures of preventive care were calculated from practice data: rates of cervical screening, cardiovascular risk assessment and HbA1c testing (which also drew on data from the national laboratory dataset).

Workforce numbers and Full Time Equivalents (FTE) came from a survey sent to practices by all participating PHOs. Data on general practitioner (GP) FTE came from 370 practices, and registered nurse (RN) FTE came from 367 practices.

### Defining practice models

- Traditional practice: Typically centred upon the GP, with mainly nursing support, operating as a small business, and owned by one or more doctors. Individual practices have a high degree of autonomy over service delivery.
- Corporate practice: A group of practices owned and run as a for-profit business entity. Corporate practices had a relatively high degree of standardisation in business and clinical processes and information technology across different sites.
- Health Care Home (HCH): the New Zealand HCH Collaborative maturity matrix focuses on business efficiency and sustainability [29]. The first practice formally enrolling in the programme in 2011. Only 14 had been fully certificated as mature HCHs by 30 September 2018 (A Maxwell, personal communication 2018).
- PHO/DHB practices: Practices owned by a PHO or a District Health Board (DHB). This was a small group that had mostly been taken over by a PHO or DHB to continue to provide primary care services in a specific location, often an underserved and/or rural area.
- Trust/NGO practices: One or more practices owned by an entity that was a not-for-profit Trust or non-governmental organisation (NGO). They had a stated purpose, identifying a health or social goal. Many were in small communities or served populations with high need.
- Māori practices: Practices owned and governed by Māori organisations, serving Māori and non-Māori patients. They were identified through lists from the Ministry of Health and DHBs together with web searches, direct contact with practices or known to investigators. There may be a small number of practices we did not identify as Māori practices.
- Pacific practices: Practices owned and governed by Pacific organisations, serving mostly Pacific and some non-Pacific patients. The same processes were used as for Māori practices (above).

Each practice was assigned to one of the seven model of care. Other practice characteristics could overlap.

### Patient health outcomes

Outcome measures were selected from existing performance indicators within national collections [30]. Measures were known to show significant inequities between groups by health need, material deprivation or ethnicity but none had previously been examined for variation by primary care model of care. The six study outcomes used were as follows.

- Polypharmacy: Patients aged 55 and older taking 5 or more long term medications over two consecutive quarters [31].
- HbA1c testing: Patients on the national VDR with one or more HbA1c test in the previous year.
- 6 Month immunisation: Children who were 6 months old at some point in the analysis period, who had received, by age 6 months, all the scheduled childhood immunisations up to and including those due at 5 months [32].
- Child ASH admissions: The number of ambulatory sensitive hospital admissions for children who were under 15 years of age at the end of the analysis period [33, 34].
- Adult ASH admissions: The number of ambulatory sensitive hospital admissions for adults who were between 45 and 64 years of age at the end of the analysis period [34].
- ED attendances: The number of attendances at an Emergency Department (ED) for each patient over the analysis period.

The analysis period was the year 1 October 2017 to 30 September 2018. All measures used the national data sets. Better outcomes were assumed to be lower polypharmacy, ASH and ED attendances, and higher HbA1c testing and childhood immunisations.

### Explanatory variables

#### Patient characteristics

These included: practice enrolled in; age, gender and ethnicity; deprivation scores (NZDep2018 and IMD); distance to the nearest ED; M3 score; having diabetes (VDR) or gout [35, 36]; being dispensed a selective serotonin reuptake inhibitor (SSRI, usually for depression), or tramadol (for moderate to severe pain), or an antibiotic. Patient changing enrolled practice in the previous year was a measure of practice continuity). Number of first medical specialist assessment (FSA) attended and not attended (did not attend, DNA) were counted for the previous year.

#### Practice characteristics

Four of the practice model names were also used to describe ownership types (Traditional, Corporate, PHO/DHB or Trust/NGO). Very Low Cost Access (VLCA) practices agree to receive increased capitation funding while limiting their fees to patients. Practices were classified as either a VLCA practice or not. Practices were designated as either urban or rural based on the rural status of a majority of their enrolled patients. The percentage of patient consultations, in the previous year and with the same GP, was used as a measure of personal continuity.

#### Primary care clinical input

Face-to-face appointments were attributed to a RN, Nurse Practitioner (NP), GP or Other. Total Consultations refers to the number of consultations with a GP or NP in the previous year (combined due to low numbers of NPs). Time spent with each patient was cumulated to a proportion of Full Time Equivalent (FTE) per 1000 enrolled patient, separately for GPs, NPs and RNs.

### Regression analyses

Multilevel mixed effects regression analyses used (only Māori) patient-level data adjusted for clustering at practice level. All analyses were conducted in R statistical software [37, 38]. Model of care categories Corporate, PHO/DHB and Trust/NGO were compared to Traditional. HCH, Māori practices and Pacific practices were compared with not-HCH, not-Māori practices and not-Pacific practices, respectively.

Regressions were interpreted to imply inequity if there was a significant association between practice models and patient health outcome after adjustment for all other factors. Statistical significance is cited at *p* ≤ 0.05, with no adjustment for repeated modelling and multiple outcomes.

## Results

At 30 September 2018 there were 988 practices with 4,561,097 patients, of which 698,924 (15.3%) were Māori. From these practices, 64 were excluded (student clinics, adolescent clinics, rest-home services or opening, closing or merging during the year of analysis). This left 924 practices with 4,491,965 patients, of which 660,752 (14.7%) were Māori, forming the study population reported here. All analyses are on enrolled patients unless specified otherwise.

### Māori patients in all practice models of care

Table 1, column 1 shows the number of practices classified to each model of care. Māori practices overlapped with other practice models, constituting 59 (of 99) Trust/NGO practices, 11 (of 127) HCH practices, 3 (of 103) Corporate practices, and 3 (of 695) Traditional practices.

**Table 1:** Māori patients, Māori population, Total population – 924 practices, by model of care

	**Māori patients in average practice**	**Māori population in primary care** *n* = 660,752	**Total population in primary care** *n* = 4,491,965
Traditional (*n* = 695)	12% (562)	59% (390,895)	73% (3,261,719)
Corporate (*n* = 103)	16% (1161)	18% (119,585)	17% (745,512)
PHO/DHB (*n* = 27)	16% (837)	3% (22,600)	3% (142,507)
Trust/NGO (*n* = 99)	37% (1290)	19% (127,672)	8% (342,226)
Māori (*n* = 65)	52% (1921)	19% (124,854)	5% (241,503)
Pacific (*n* = 15)	10% (321)	1% (4,816)	1% (48,233)
HCH (*n* = 127)	15% (1043)	20% (132,448)	20% (909,690)

Percentages do not add to 100 because models of care overlap

A total of 124,854 Māori patients were enrolled in 65 Māori practices, constituting 19% of the total enrolled Māori population. Traditional practices enrolled 59% of Māori and 73% of the total population enrolled in general practice.

On average, 52% of patients in a Māori practice were Māori, three to four times the percentage of other practice types except for Trust/NGO practices which overlap extensively with Māori practices. Traditional practices had the lowest average percentage of Māori patients at 12%.

Comparing patient-related characteristics between the Māori population and the total population shows a number of substantive differences (Table 2). The Māori population included a higher percentage of children and young people and a lower percentage of older adults. Twice as many Māori lived in Quintile 5 (Q5) areas (highest deprivation), reflected in a higher median IMD. A much higher percentage of Māori enrolled in VLCA practices, with lower patient fees. More Māori lived rurally (21.5%) compared to the total population (15%), with a greater distance to the nearest ED.

**Table 2:** Patient-related characteristics, Māori patients and total population

	**Māori patients** *n* = 660,752	**Total population** *n* = 4,491,965
**Patient age**		
Age ≤ 14	32.2%	20.4%
Age ≥ 65	6.4%	16.0%
**Deprivation**		
Quintile 5	42.8%	19.4%
Index of Multiple Deprivation (IMD) ^a^	4535 (3038 – 5431)	2981 (1463 – 4505)
Enrolled in VLCA practice	55.8%	30.4%
**Conditions & management**		
Dispensed antibiotic in last year	44.5%	39.9%
Dispensed tramadol in last year	5.3%	5.1%
Dispensed SSRI in last year	4.7%	6.8%
Diabetes	5.9%	5.4%
Average Multimorbidity (M3)	0.151	0.132
First Specialist Assessment (FSA) ^b^	7.6%, 1.3%, 0.4%	8.1%, 1.5%, 0.5%
FSA not attended in last year ^b^	1.19%, 0.25%, 0.06%	0.57%, 0.09%, 0.02%
Continuity of practice	74.6%	76.8%
**Rurality & distance**		
Patient distance to nearest ED (km) ^a^	6.9 (3.3 – 19.7)	6.8 (3.5 – 16.6)
Patient rural residential address	21.5%	15.0%
**RN & GP time**		
RN hours / 1000 enrolled patients ^a^	0.11 (0 – 0.52)	0.09 (0 – 0.49)
GP hours / 1000 enrolled patients ^a^	0.46 (0.22 – 0.88)	0.48 (0.22 – 0.88)

^a^ median (25th–75th centile)

^b^ % at 1, 2 and 3 or more FSA attended or not attended

Compared with the total population, Māori patients were more likely to be dispensed antibiotics or tramadol; to have diabetes and a higher M3 score; and were less likely to be dispensed SSRIs.

Māori had close to the same rate of FSA appointments made (attended plus non-attended) but were more than twice as likely to not attend a FSA. There was a small decrease in continuity of practice for Māori.

The median FTE that RNs spent with a Māori patient was 22% higher than for the total population (0.11 FTE per 1000 patients compared to 0.09 FTE). By the same measure, GP FTE was 9% lower for a Māori patient.

### Māori practices—a model of care

Māori practices show distinct differences from all practices combined (Total practices), see Table 3. Māori practice size was smaller, with more younger and fewer older patients; there was a much larger percentage of Māori patients and a much smaller percentage of non-Māori non-Pacific patients. Māori practices were much more likely to hold a VLCA contract.

**Table 3:** Māori practices and Total practices: profile of patient populations

	**Māori practices** *n* = 65	**Total practices** *n* = 924
**Patient number, age, ethnicity**		
N patients enrolled ^a^	2954 (1528—4975)	3622 (2074—6189)
Age ≤ 14 ^a^	24.8% (22.1—27.5)	19.4% (16.9—22.4)
Age ≥ 65 ^a^	10.8% (7.7—14.5)	16.4% (11.6—21.1)
Māori patients ^a^	61.8% (34.9 – 77.3)	9.5% (5.7—17.1)
Pacific patients ^a^	3.5% (1.8 – 8.5)	2.0% (1.1—5.0)
Non-Māori non-Pacific patients ^a^	28.6% (16.8 – 54.6)	87.0% (72.5—91.9)
**Level of deprivation**		
Quintile 5 ^a^	58.4% (38.2—71.9)	11.7% (4.8—29.1)
Index of Multiple Deprivation (IMD) ^a^	4568 (3987—5030)	2882 (2191—3775)
VLCA	93.9%	29.7%
**Conditions & management**		
Dispensed SSRI in last year ^a^	4.4% (3.1—6.5)	7.0% (5.2—8.5)
Dispensed antibiotic in last year ^a^	41.9% (36.6—45.0)	39.9% (35.3—44.5)
Diabetes ^a^	7.6% (6.7—8.7)	5.2% (4.0—6.8)
Multimorbidity index (M3) ^a^	0.18 (0.16—0.21)	0.14 (0.11—0.16)
First Specialist Assessment (FSA) attended in last year ^b^	0.13 (0.11—0.16)	0.12 (0.11—0.15)
FSA not attended in last year ^b^	0.02 (0.01—0.03)	0.01 (0—0.01)
Continuity of practice ^a^	73.3% (66.7—79.2)	79.2% (73.0—83.5)
**Rurality & distance**		
Practice distance to nearest ED (km) ^a^	8.7 (5.9—28.4)	7.4 (5.6—16.1)
Practice rural	33.9%	16.8%
**RN & GP time**		
RN hours / 1000 enrolled patients ^a^	0.79 (0.68—0.97)	0.54 (0.38—0.72)
GP hours / 1000 enrolled patients ^a^	0.68 (0.55—0.9)	0.63 (0.51—0.79)
**Preventative care**		
Cervical screening / 1000 women	394	341
Cardiovascular risk assessment / 1000 adults	238	211
HbA1c testing / 1000 adults with diabetes	1630	1890

^a^ number or percentage median (25th–75th centile)

^b ^median FSA attended or not attended per person (25th – 75th centile)

The profile of the patient population in Māori practices suggests a higher level of need for primary care than in Total practices. Patients in Māori practices were more likely to live in material deprivation, more likely to be dispensed an antibiotic, less likely to be dispensed an SSRI, more likely to have diabetes, a higher M3, an FSA, not attend an FSA and less likely to have continuity of practice.

Māori practices were twice as likely to be rural (33.9% compared to 16.8%). Distance from the practice to the nearest ED was considerably greater for Māori practices.

Patients in Māori practices were a little more likely than those in Total practices to attend a FSA, and twice as likely to not attend a booked FSA.

Considerably more RN (46%) and GP (8%) FTE was spent with patients in Māori practices than in Total practices. Cervical smear rates and cardiovascular risk assessment rates were higher, and HbA1c test rates were lower than in Total practices.

### Associations of models of care with patient health outcomes

The final regression models include only Māori patients, with practice models of care entered as explanatory variables, shown in Tables 4 and 5. The bulk of variance was at patient level. The proportion of variance at practice level was 2% for polypharmacy, 8% for HbA1c testing, 8% for immunisations, 7% for child ASH, 6% for adult ASH, and 21% for ED attendance. In the text that follows, percentages are median predicted probabilities of outcomes for polypharmacy, HbA1c testing and immunisations. Numbers are median predicted rates per 1000 patients for outcomes child ASH, adult ASH and ED attendances.

- Compared with being enrolled in a Traditional practice
  - ◦ Corporate practice enrolment was associated with
    - ▪ 26.3% (4.4% less) polypharmacy (Traditional 29.7%)
    - ▪ 93 (16 more) adult ASH (Traditional 77)
  - ◦ PHO/DHB practice enrolment was associated with
    - ▪ 116 (49 more) adult ASH (Traditional 77)
    - ▪ 424 (59 more) ED attendances (Traditional 365)
  - ◦ Trust/NGO practice enrolment was associated with
    - ▪ 62 (18 more) child ASH (Traditional 44)
    - ▪ 106 (29 more) adult ASH (Traditional 77)
    - ▪ 460 (95 more) ED attendances (Traditional 365)
- Māori practice enrolment was associated with
  - ◦ 26.2% (3.7% less) polypharmacy (other practices 29.9%)
  - ◦ 53.0% (13.4% less) immunisations (other practices 66.4%)
- Pacific practice enrolment was associated with
  - ◦ 35.5% (29.0% less) immunisation (other practices 64.5%)
  - ◦ 36 (41 fewer) adult ASH (overall mean 77)
  - ◦ 283 (119 fewer) ED attendances (overall mean 365)
- HCH practice enrolment was associated with
  - ◦ 27.8% (1.6% less) polypharmacy (other practices 29.4%)
  - ◦ 69.2% (6.2% more) immunisations (other practices 63.0%)
  - ◦ 342 (23 fewer) ED attendances (overall mean 365)

**Table 4:** Predicted probability of outcomes for each variable. Final models for each primary outcome for Māori enrolled in 924 practices

	**Polypharmacy in people age ≥ 55 R**^**2**^** = 0.4466 *N* = 56,280**	**HbA1c in people with diabetes R**^**2**^** = 0.1755 *N* = 21,586**	**Immunisations at age 6 months R**^**2**^** = 0.0811 *N* = 4,775**	**Child ASH per 1000 children R**^**2**^** not applicable *N* = 118,889**	**Adult ASH per 1000 enrolled adults R**^**2**^** not applicable *N* = 73,950**	**ED attendances per 1000 enrolled patients R**^**2**^** not applicable *N* = 366,486**
**Overall mean**	33.4%	80.9%	63.6%	44	77	365
**Variable (Comparator, blank if continuous variable)**						
***Practice models***						
**Corporate (Traditional)**	26.3% (29.7%) **	81.8% (83.8%)	61.8% (64.9%)	-9.4%	21.2% *	-2.1%
**PHO/DHB (Traditional)**	26.3% (29.2%)	80.2% (83.6%)	57.0% (64.6%)	-2.7%	51.0 **	16.2% *
**Trust/NGO (Traditional)**	29.3% (29.0%)	84.5% (83.1%)	68.2% (63.6%)	41.1% *	37.2% **	26.0% ***
**HCH Practice (All others)**	27.8% (29.4%) *	83.4% (83.5%)	69.2% (63.0%) **	5.5%	-4.9%	-6.3% *
**Māori Practice (All others)**	26.2% (29.8%) *	80.8% (84.3%)	53.0% (66.4%) **	-10.1%	-12.9%	-4.8%
**Pacific Practice (All others)**	25.3% (29.1%)	84.5% (83.5%)	35.5% (64.5%) **	-33.6%	-52.9% **	-22.4% *
***Patient characteristics***						
**Male (Female)**		83.5% (83.4%)			15.1% ***	
**Quintile 5 (Not Q5)**		83.0% (83.9%)				
**IMD Score** **(25**^**th**^**, 50**^**th**^**, 75**^**th**^** centiles)**	27.6% *** 28.5% 29.4%		66.4% *** 63.2% 61.2%	-6.6% *** 2.8% 8.8%	-7.5% *** 4.2% 11.9%	-5.0% *** 2.2% 6.8%
**Diabetes (No diabetes)**	58.6% (21.9%) ***				12.6% **	
**Gout (No gout)**	54.5% (25.2%) ***	86.3% (82.6%) ***			9.4%	
**HbA1c (No HbA1c)**	32.3% (24.7%) ***					
**SSRI (No SSRI)**	52.5% (27.7%) ***					29.8% ***
**Antibiotic (No antibiotic)**	32.4% (26.3%) ***	85.2% (81.2%) ***		129.1% ***	163.9% ***	144.1% ***
**Tramadol (No tramadol)**					58.3% ***	85.3% ***
**M3** **(25**^**th**^**, 50**^**th**^**, 75**^**th**^** centiles)** **(ASH&ED ref: M3 = 0)**	25.5% *** 28.3% 34.0%	85.4% *** 84.2% 83.0%		49.7% *** 188.7% 421.9%	22.1% *** 69.0% 126.6%	15.6% *** 46.4% 81.1%
**Continuity of practice (No continuity)**				-13.4%***	-23.5% ***	-26.3% ***
**Distance﻿ to Nearest ED** **(Immunisation rate at 1, 20, 100 km; ASH&ED ref: average distance)**			64.9% 64.5% 60.9%		4.2% * -1.2% -21.3%	8.3% *** -2.9% -38.7%
**First Specialist Assessment** **(1, 2, 3)**	30.3% *** 31.9% 33.6%	84.0% 84.8% 85.6%		42.5% *** 102.9% 189.0%	32.2% *** 74.7% 130.8%	34.4% *** 80.6% 142.8%
**First Specialist Assessment –** **Did Not Attend** **(1, 2, 3)**			60.2% 55.8% 51.3%	9.7% 20.4% 32.0%	66.1% *** 176.0% 358.4%	44.7% *** 109.4% 203.1%
***Practice characteristics***						
**VLCA (not VLCA)**		83.0% (84.3%)	62.6% (66.0%)			
**Urban Practice (rural)**	29.2% (28.9%)	83.29% (84.0%)	64.6% (63.1%)	-4.5%	-7.3%	
**Continuity of GP** **(25**^**th**^**, 50**^**th**^**, 75**^**th**^ **centiles)**				-5.7% -7.6% -11.2%		
***Clinician input***						
**Total Consultations** **(1, 2, 4)**	19.3% *** 21.9% 27.7%	77.1% *** 78.7% 81.6%	62.3% 62.8% 63.9%	7.4% *** 15.4% 33.2%	5.3% *** 10.8% 22.8%	7.1% *** 14.6% 31.3%
**Nurse FTE** **(25**^**th**^**, 50**^**th**^**, 75**^**th**^ **centiles)**		82.5% *** 82.7% 83.2%	62.5% ** 62.8% 63.9%			
**GP FTE** **(25**^**th**^**, 50**^**th**^**, 75**^**th**^ **centiles)**			63.7% 64.1% 64.2%	-1.82% -0.36% -0.09%		
***Interactions***						
**Total Consults X Corporate** **1 more consult (Traditional)**	4.6% (3.3%) ***	1.3% (1.3%)	-0.33% (0.51%)	10.5% (7.4%) *	4.4% (5.3%)	8.0% (7.1%) **
**Total Consults X PHO/DHB** **1 more consult (Traditional)**	4.9% (3.3%) *	1.6% (1.3%)	0.97% (0.51%)	9.1% (7.4%)	7.3% (5.3%)	8.8% (7.15%) *
**Total Consults X Trust/NGO ** **1 more consult (Traditional)**	4.1% (3.3%) *	1.2% (1.3%)	-0.01% (0.51%)	9.5% (7.4%)	5.1% (5.3%)	7.2% (7.1%)
**Nurse FTE X Corporate** **1 more hour (Traditional)**		0.4% (1.34%)	-1.10% (2.7%)			
**Nurse FTE X PHO/DHB** **1 more hour (Traditional)**		1.4% (1.3%)	2.6% (2.7%)			
**Nurse FTE X Trust/NGO**n **1 more hour (Traditional)**		0.7% (1.3%) *	-2.9% (2.8%)			
**GP FTE X Corporate** **1 more hour (Traditional)**			1.6% (0.7%)	-8.% (2.7%) **		
**GP FTE X PHO/DHB** **1 more hour (Traditional)**			-0.05% (0.74%)	-0.29% (2.72%)		
**GP FTE X Trust/NGO** **1 more hour (Traditional)**			-0.76% (0.74%)	-6.1% (2.7%) *		
**Continuity of GP X Corporate**				-1.6% (-1.2%)		
**Continuity of GP X PHO/DHB**				-0.2% (-1.2%)		
**Continuity of GP X Trust/NGO**				-2.4% (-1.2%)		

Description of final regression models

• The polypharmacy regression used a logistic regression model. The dependent variable was polypharmacy, which took value 1 if a person was taking five or more drugs and 0 otherwise. We allowed for random intercepts and random slopes on M3 and Total Contacts

• The HbA1c test regression used a logistic regression model. The dependent variable was HbA1c test, which took value 1 if a person had an HbA1c test within a year and 0 otherwise. We allowed for random intercepts and random slopes on M3, Total Contacts, and Nurse FTE

• The 6 month immunisations regression used a logistic regression model. The dependent variable was 6 month immunisations, which took value 1 if a child had all their required immunisations (according to the vaccine schedule) by six months of age and 0 otherwise. We allowed for random intercepts and random slopes on Total Contacts, GP FTE, and Nurse FTE

• The child ASH admissions regression used a negative binomial regression model. The dependent variable was a patient’s number of ASH admissions over the period of analysis. We allowed for random intercepts and random slopes on Total Contacts, GP FTE, and Percent Main Provider

• The adult ASH admissions regression used a negative binomial regression model. The dependent variable was a patient’s number of adult ASH admissions over the period of analysis. We allowed for random intercepts and random slopes on Total Contacts and M3

• The ED attendances regression used a negative binomial regression model. The dependent variable was a patient’s number of ED attendances over the period of analysis. We allowed for random intercepts and random slopes for Total Contacts and M3

Blank cells indicate variables not retained in final models

* *p* < 0.05

** *p* < 0.01

*** *p* < 0.001, 0

*** *p* < 1e^−16^

**Table 5:** Regression coefficients, final models for each primary outcome, Māori in 924 practices

	**Polypharmacy in people age ≥ 65** R^2^ = 0.4466 *N* = 56,280	**HbA1c in people with diabetes** R^2^ = 0.1755 *N* = 21,586	**Immunisations at age 6 months** R^2^ = 0.0811 *N* = 4,775	**Child ASH** R^2^ not applicable *N* = 118,889	**Adult ASH** R^2^ not applicable *N* = 73,950	**ED attendances** R^2^ not applicable *N* = 366,486
**Overall mean**	33.4%	80.9%	63.6%	44 per 1000 enrolled children	77 per 1000 enrolled adults	365 per 1000 enrolled patients
	Estimate *p* value	Estimate *p* value	Estimate *p* value	Estimate *p* value	Estimate *p* value	Estimate *p* value
**Intercept**	-2.203534	1.105886	0.584101	-4.00084	-4.13696	-2.109394
	0 ***	0 ***	3e-06 ***	0 ***	0 ***	0 ***
***Practice models***						
** Corporate ** ^**a**^	-0.165781	-0.142365	-0.13325	-0.09902	0.191857	-0.020883
	0.009601 **	0.195163	0.36815	0.462772	0.013186 *	0.577196
** PHO/DHB ** ^**a**^	-0.146741	-0.227092	-0.31994	-0.02732	0.411895	0.150022
	0.220088	0.281985	0.254857	0.913146	0.005077 **	0.040814 *
** Trust/NGO ** ^**a**^	0.011866	0.097556	0.205518	0.344206	0.316441	0.23071
	0.889102	0.564796	0.349084	0.021422 *	0.004577 **	9e-05 ***
** HCH Practice ** ^**b**^	-0.080916	-0.004833	0.277053	0.053086	-0.05016	-0.06501
	0.035649 *	0.954487	0.003089 **	0.306358	0.375549	0.040928 *
** Māori Practice ** ^**b**^	-0.182819	-0.241891	-0.56065	-0.10639	-0.13757	-0.048903
	0.014506 *	0.143095	0.003662 **	0.302633	0.18591	0.425907
** Pacific Practice ** ^**b**^	-0.190856	0.080802	-1.19511	-0.40935	-0.75323	-0.253206
	0.267265	0.786684	0.009291 **	0.076001	0.003492 **	0.01661 *
***Patient factors***						
** Age 00–04**		-3.595615		0.22549		0.508938
		1.2e-05 ***		0 ***		0 ***
** Age 05–09**		-4.455915		reference		-0.041319
		0 ***				0.045345 *
** Age 10–14**		-2.319399		-0.96348		0.031559
		0 ***		0 ***		0.137948
** Age 15–19**		-1.432507				0.462977
		0 ***				0 ***
** Age 20–24**		-1.139149				0.582737
		0 ***				0 ***
** Age 25–29**		-0.875194				0.422451
		0 ***				0 ***
** Age 30–34**		-0.59253				0.282423
		0 ***				0 ***
** Age 35–39**		-0.408235				0.167026
		3.2e-05 ***				0 ***
** Age 40–44**		-0.128773				0.079423
		0.156121				0.000559 ***
** Age 45–49**		Reference			-0.1387	Reference
					0.007081 **	
** Age 50–54**		0.14116			Reference	-0.042569
		0.075067				0.064319
** Age 55–59**	-1.181874	0.354513			0.01749	-0.14237
	0 ***	5e-06 ***			0.720436	0 ***
** Age 60–64**	-0.819014	0.528367			-0.00722	-0.160126
	0 ***	0 ***			0.889479	0 ***
** Age 65–69**	-0.480979	0.462988				-0.212001
	0 ***	0 ***				0 ***
** Age 70–74**	-0.212458	0.545326				-0.174538
	3.3e-05 ***	0 ***				0 ***
** Age 75–59**	Reference	0.446933				-0.074983
		2.2e-05 ***				0.033079 *
** Age 80–84**	0.022677	0.197183				-0.020303
	0.743895	0.11547				0.645975
** Age 85 + **	0.232713	-0.104139				0.207933
	0.005858 **	0.513466				6.7e-05 ***
** Male ** ^**c**^		0.011643			0.140714	
		0.763229			0.000169 ***	
** Quintile 5 ** ^**c**^		-0.066351				
		0.104868				
** IMD ** ^**d**^	0.045574		-0.15201	0.101755	0.12766	0.076927
	0.000344 ***		2.3e-05 ***	0 ***	0 ***	0 ***
** Diabetes ** ^**c**^	1.61725				0.118262	
	0 ***				0.006907 **	
** Gout ** ^**c**^	1.268493	0.27879			0.089678	
	0 ***	0 ***			0.111175	
** HbA1c test ** ^**c**^	0.375364					
	0 ***					
** SSRI ** ^**c**^	1.059998					0.260437
	0 ***					0 ***
** Tramadol ** ^**c**^					0.459292	0.892234
					0 ***	0 ***
** Antibiotics ** ^**c**^	0.2975	0.291107		0.829183	0.970391	0.616753
	0 ***	0 ***		0 ***	0 ***	0 ***
** M3 ** ^**d**^	0.660593	-0.264025		1.896788	0.939156	0.681459
	0 ***	0 ***		0 ***	0 ***	0 ***
** Continuity of practice ** ^**c**^				-0.14405	-0.26749	-0.305727
				2.4e-05 ***	0 ***	0 ***
** First Specialist Assessment ** ^**d**^	0.076018	0.060579		0.353798	0.2789	0.295668
	0.000108 ***	0.056068		0 ***	0 ***	0 ***
** FSA Did Not Attend ** ^**d**^			-0.18188	0.092618	0.507525	0.369589
			0.170847	0.23251	0 ***	0 ***
***Practice factors***						
** Urban ** ^**c**^	0.011164	-0.054702	0.065662	-0.04651	-0.07605	
	0.753151	0.503673	0.552787	0.372884	0.23598	
** VLCA** ^**c**^		-0.098921	-0.14957			
		0.177128	0.081755			
** Continuity of GP ** ^**d**^				-0.11821		
				0.135424		
** Distance to nearest ED ** ^**d**^			-0.02875		-0.05262	-0.102692
			0.483513		0.037277 *	0 ***
***Primary care clinical input***						
** NP + GP consults ** ^**d**^	0.15582	0.09311	0.022251	0.071637	0.051258	0.068116
	0 ***	0 ***	0.059271	0 ***	0 ***	0 ***
** RN hours ** ^**d**^		0.14258	0.118932			
		0.000643 ***	0.009092 **			
** GP hours ** ^**d**^			0.338837	0.031598		
			-0.00266	0.091842		
***Interactions***						
** NP + GP consults X Corporate ** ^**a**^	0.04552	0.010572	-0.03608	0.027746	-0.00844	0.009074
	0.000226 ***	0.592731	0.160883	0.018895 *	0.36928	0.001684 **
** NP + GP consults X PHO/DHB ** ^**a**^	0.056796	0.050163	0.022056	0.015166	0.019328	0.016385
	0.022128 *	0.247839	0.687908	0.596488	0.360744	0.010759 *
** NP + GP consults X Trust/NGO ** ^**a**^	0.027916	-0.00713	-0.02301	0.018628	-0.00218	0.001728
	0.012868 *	0.689318	0.376409	0.104953	0.797324	0.535464
** RN hours X Corporate ** ^**a**^		-0.105067	-0.16496			
		0.262851	0.119132			
** RN hours X PHO/DHB ** ^**a**^		0.006266	-0.00852			
		0.965215	0.954599			
** RN hours X Trust/NGO ** ^**a**^		-0.067274	-0.23994			
		0.404552	0.017736 *			
** GP hours X Corporate ** ^**a**^			0.051979	-0.12916		
			0.585034	0.002464 **		
** GP hours X PHO/DHB ** ^**a**^			-0.04751	-0.03497		
			0.807839	0.705262		
** GP hours X Trust/NGO ** ^**a**^			-0.0895	-0.10583		
			0.372925	0.010701 *		
** Continuity of GP X Corporate ** ^**a**^				-0.03881		
				0.811535		
** Continuity of GP X PHO/DHB ** ^**a**^				0.094332		
				0.768363		
** Continuity of GP X Trust/NGO ** ^**a**^				-0.12182		
				0.432376		

Blank cells indicate variables not retained in final models

^*^ p < 0.05

^**^ p < 0.01

^*** ^*p* < 0.001, 0

^***^ p < 1e^−16^

^a^ reference is Traditional practice

^b^ reference is all other practices

^c^ dummy variable

^d^ continuous variable

### Associations of patient characteristics with patient health outcomes

The following statements apply to the average patient. There were no statistically significant associations with: VLCA, continuity of GP, Quintile 5 deprivation (tested only in respect of HbA1c testing; all other outcomes were significantly associated with IMD), urban/rural and GP hours.

For IMD, increasing deprivation from the 25th to the 75th centile was associated with changes of 5.2% absolute lower immunisations, 15.4% higher child ASH, 19.4% higher adult ASH and 11.8% higher ED attendances.

For M3, increasing multimorbidity from the 25th to the 75th centile was associated with absolute changes of 2.4% lower immunisations (from 85.4% to 83.0%), 372.2% more child ASH (from 22 to 186), 104.5% more adult ASH (from 17 to 97) and 65.5% more ED attendances (from 57 to 296).

Continuity of practice was associated with relative changes in child ASH (down 13.4%), adult ASH (down 23.5%) and ED attendances (down 26.3%), compared with no continuity (i.e. patients changing practice within a year).

- Age was strongly associated with all outcomes (except immunisations, which were measured at a specific age)
- Being male was associated with more adult ASH
- Higher IMD was associated with more polypharmacy, fewer immunisations, and more child ASH, adult ASH and ED attendances
- Having diabetes was associated with more polypharmacy and adult ASH
- Having gout was associated with more polypharmacy and HbA1c testing
- HbA1c testing was associated with more polypharmacy
- SSRI dispensing was associated with more polypharmacy and ED attendances
- Tramadol dispensing was associated with more adult ASH and ED attendances
- Antibiotic dispensing was associated with more polypharmacy, HbA1c testing, child ASH, adult ASH and ED attendances
- Higher M3 score was associated with polypharmacy and more child ASH, adult ASH and ED attendances
- First Specialist Assessment was associated with more polypharmacy, child ASH, adult ASH and ED attendances
- First Specialist Assessment Did Not Attend was associated with more adult ASH and ED attendances
- Patient distance to nearest ED was associated with more adult ASH and ED attendances

### Associations of primary care clinical input with patient health outcomes

- NP + GP consultation count was associated with more polypharmacy, HbA1c testing, child ASH, adult ASH and ED attendances
- RN hours were associated with more HbA1c testing and more immunisations
- Associations with an additional NP or GP consultation, compared with being enrolled in a Traditional practice: enrolment in a Corporate practice was associated with more polypharmacy, child ASH and ED attendances; enrolment in a PHO/DHB practice was associated with more polypharmacy and ED attendances; enrolment in a Trust/NGO practice was associated with more polypharmacy
- An additional hour with a RN, in a Trust/NGO practice, was associated with fewer HbA1c tests than in a Traditional practice
- An additional hour with a GP, in a Corporate practice or a Trust/NGO practice, was associated with fewer child ASH than in a Traditional practice

## Discussion

Māori practices are an expression of the rights of Māori to actively lead health and social service provision in the face of enduring health system failure. Mostly, patient health outcomes for Māori patients at Māori practices were not different from other models of care, despite the high health risk profile of their enrolled patients. Across all models of care, the high primary care needs of many Māori patients remain unmet, despite increased clinical input.

### Patient health outcomes

Outcomes for Māori patients in Māori practices were not different from outcomes in other practice models with respect HbA1c testing, ambulatory sensitive hospitalisations or ED attendance. They had a slightly lower rate of polypharmacy (by 3.7 percentage points) and lower childhood immunisations completed by age 6 months (by 13.4 percentage points).

Polypharmacy was lower in Māori, Corporate and HCH practices. At a population level, lower polypharmacy is considered a marker of good primary care even though the ideal number of dispensed medications for any one person, and the ideal percentage of polypharmacy for a practice, are unknown. Polypharmacy was measured using dispensing data, not prescribing data, and Māori patients do not pick up prescribed medication as often as non-Māori due to cost (Māori children about 7.9 times and Māori adults about 3.3 times more likely than non-Māori) [39]. Furthermore, Māori are known to be under-prescribed against guideline standards for several conditions including gout [40] and diabetes [41].

Rates of completed immunisations at age 6 months were lower, for Māori children, in Māori and Pacific practices, and higher in HCH practices. Our primary outcomes paper also showed that Māori children were less likely to be immunised by age 6 months than non-Māori, adjusted for all other factors including model of care. Some of the overdue immunisations would have been delivered after the recommended age, but still represent prolonged vulnerability to vaccine-preventable diseases. Since this study, immunisation rates have dropped dramatically; the rate for Māori children in South Auckland is now “dire” at 34% [42]. In part this has been due to shifting resources to address the COVID-19 pandemic and must now be addressed as a public health emergency.

ED attendance showed the largest variance (21%), at practice level, in the regressions. For Māori patients, ED attendance rates were higher in PHO/DHB and Trust/NGO practices and lower in Pacific and HCH practices.

### Māori practices

The 65 Māori practices enrolled 124,854 Māori patients (19% of all Māori). In the average Māori practice, 52% of patients were Māori, more than four times the percentage of Māori in Traditional practices and three times the percentage in Health Care Homes and Corporate practices.

### Rural practices and populations

Twice as many Māori practices were rural (34%) than for all practices (17%) and one third more Māori lived rurally (21.5%) than for the total population (15%). Absence of a ‘base’ hospital in a locality is part of the definition of rurality [43], so the distribution of Māori populations and practices has implications for accessing specialist health services and for patient health outcomes. While higher rural mortality rates have not been identified for Māori in Aotearoa New Zealand in previous Ministry of Health reports, a recent study found rural Māori experience greater all-cause mortality (Standardised Incident Rate Ratio (SIRR) 1.07) and amenable mortality (SIRR 1.13) than their urban peers, which increased as rurality increased [44]. This is consistent with higher rural mortality rates seen for Indigenous populations in Australia and the United States [45].

A high percentage of clinical work, in Māori practices, was undertaken by nurses, reflected in the ratio of nurses to doctors; this is discussed further in an accompanying paper on nurses’ work.

### Māori patients in all practices

Traditional practices have, on average, a relatively low risk patient population and a relatively low percentage of Māori patients (12%). It is possible that such practices can apply resources to provide more clinical input to those with high needs. In addition, clinicians, their organisations and the health system “need to be engaged in working towards cultural safety and critical consciousness”. They must be prepared to critique power structures and “challenge their own culture and cultural systems rather than prioritise becoming ‘competent’ in the cultures of others” [46]. Cultural safety is about recognising the barriers to clinical effectiveness that arise from power imbalance between provider and patient [47]. It is not about clinicians learning the cultural customs of different ethnic groups, but rather cultural safety aims to improve care through awareness of difference, decolonising, considering power relationships, and by allowing patients to determine whether clinical encounters are safe [47, 48]. Cultural safety (kawa whakaruruhau within a Māori context) is necessary to achieve positive patient health outcomes for Māori patients and whānau [49].

The study included 660,752 Māori patients who comprised about 50% more children and young people and less than half the percentage of older adults than are present in the total population. There is a raised burden of care provision because Māori children and Māori adults collectively are known to have high rates of illness. For example, ASH rates for Māori 0–4 year olds are more than 1.5 times the rate for non-Māori non-Pacific children [50]. The most common conditions for which Māori children are hospitalised are asthma and wheeze, dental procedures, acute upper respiratory infections, gastroenteritis, skin infections and pneumonia. Māori adults, compared to the general population, have higher rates of cardiovascular disease, stroke, diabetes, cancer and respiratory diseases, with higher rates of complications generally reflecting onset at an earlier age [8]. Life expectancy is reduced and leading causes of death and years of life lost are heart disease, stroke, chronic lung disease, lung cancer (women and men), breast cancer (women), suicide (men), diabetes, and motor vehicle accidents [51].

Twice as many Māori lived in Quintile 5 areas (highest deprivation), compared to the total population. This was reflected in higher Māori scores for the Index of Multiple Deprivation (IMD), which includes measures of employment, income, crime, housing, education and access. Nearly twice as many Māori, compared to the total population, were enrolled in practices with a Very Low Cost Access contract, usually chosen by patients for their lower fees. Māori also had higher levels, compared to the total population, of morbidity and multi-morbidity as indicated by the Measuring Multimorbidity Index (scored across more than 50 serious health conditions), rates of diabetes and gout, and rates of prescribing for antibiotics and tramadol. Taken together, these factors point to a population that has high health needs and clinical complexity, and who can be reasonably expected to need substantial health and social services to approach equitable patient outcomes.

Despite Māori adults being about 1.5 times as likely as non-Māori to report a high or very high probability of having an anxiety or depressive disorder [8], rates of dispensing SSRIs were lower for Māori. This may indicate reluctance to disclose mental illness, under-diagnosis or under-prescribing for depression [52].

Higher rates of antibiotic dispensing may be an appropriate response to rates of rheumatic fever, respiratory infections and skin infections [8], although achieving equity in these patient outcomes requires also addressing social determinants of health. Māori practices often access funding, for example, for housing, with nurses and community health workers / kaimahi working at the intersection of health and social care [53–55].

Higher rates of tramadol may reflect increased need for analgesia. For example, Māori aged 15–64 years have an unintentional injury mortality rate more than 1.5 times that for non-Māori and the unintentional injury hospitalisation rate for Māori was about 30% higher than that of non-Māori [8].

### Clinical input

The median FTE that RNs spent with a Māori patient was 22% higher than for the total population (0.11 FTE patient compared to 0.09 FTE). GP FTE was 4% lower for Māori than for the total population. Given the high health needs in this population, these rates appear too low to achieve equity of patient health outcomes.

The rate of FSA appointments was close to total population, however, given the higher health needs of Māori, a higher rate of appointments would seem appropriate. The rate of non-attendance remains concerning. FSA referrals are generated from both primary and secondary care so more information is required to investigate what might be low referral rates.

### Health system reforms

The New Zealand Government has acknowledged the health reforms of 2001 failed to improve primary health care services and health outcomes, especially for Māori [1]. Currently, structural reform of the health system is in process and many Māori welcome the potential of Te Aka Whai Ora – Māori Health Authority. However, most of the functions of Te Aka Whai Ora existed already and government entities already had legislative and regulatory abilities to perform many of these roles, but these were not prioritised. Racism exists within structures, policies, practices, norms and values which permit racist outcomes and “prevent us acting to overcome them” [9]. Equitable outcomes cannot be achieved without a culture change within the health system that prioritise equity and complies with guarantees of Te Tiriti o Waitangi.

### Limitations

Each practice had its own history of adapting to their enrolled patient population, region, and policy and funding context. Grouping them together into “models” was a necessary simplification to address our research question. We have assumed that, at a system level, a lower rate is better for polypharmacy, ASH and ED attendances, and a higher rate is better for HbA1c testing and immunisations, but acknowledge that the best outcome for individual patients remains unknown.

Associations from cross-sectional analysis cannot prove causality, and many factors affecting patient health outcomes reside outside primary care. Although trends over time for each outcome may have been more informative, this come with its own difficulties; patient turnover within practices, practices opening, closing or merging during the year studied, and periodic changes to practice funding policies.

Although explanatory variables were divided into three categories – patient characteristics, practice characteristics and clinical input, some factors fall into more than one category.

When describing preventive care we did not have the data to identify eligible populations meeting the complex recommendations of guidelines. While our numerators are accurate, denominators likely included persons who were not eligible, making calculated rates lower than a “true” measure. However, since the same method is applied to all practice models, relative differences between models are assumed to remain valid.

## Conclusions

In Aotearoa New Zealand there is a unique discourse because of Te Tiriti o Waitangi partnership. Māori practices, delivering primary care to Māori and non-Māori patients, are an expression of Māori self-determination. Government funding should support under-resourced Māori practices and ensure accountability for the health outcomes of Māori patients in all models of general practice. A cultural reform is needed to move the health and disability sector to be pro-equity, culturally safe, Tiriti compliant and anti-racist.

## Data Availability

Data for this project were collected on condition of anonymity of patients, practices and PHOs, with an agreement to delete data once the purpose of the project was met. Data collected from practices and PHOs is not available. Data collated from national data sets is available in summary form on request to author TK (t.kenealy@auckland.ac.nz).

## Abbreviations

DHB: District Health Board; responsible for public-funded health services, including hospitals, in a geographical area; ceased to exist as independent entities from July 2022
ED: Emergency department
FSA: First specialist assessment
FSA DNA: Did not attend first specialist assessment
FTE: Full time equivalent; refers to hours of work
GP: General practitioner; family physician
HCH: Health care home
IMD: Index of multiple deprivation
M3: Measuring Multi-morbidity index
NGO: Non-governmental organisation
NP: Nurse Practitioner
PHO: Primary Health Organisation; all general practices belong to a PHO, which contracts to a DHB to provide primary care services
Q5: Quintile 5 (most deprived) on NZ Deprivation Index
RN: Registered nurse
SSRI: Selective serotonin re-uptake inhibitor; a widely used category of antidepressant
VDR: Virtual diabetes register; a national list of people considered to have diabetes
VLCA: Very low cost access; practice with higher subsidies and lower fees

## Footnotes

1 Renwick [20] explains that by the time treaties were signed on Vancouver Island, BC, Canada—a mere decade later—“British imperial policy was determined by strategic considerations not humanitarian intentions”. Wickliffe [19] asserts that “The Treaty of Waitangi is fundamentally different to treaties in the Americas … [which] did not deal with the sovereign status of indigenous polities”.

2 The Waitangi Tribunal is a standing commission of inquiry. It makes recommendations on claims brought by Māori relating to legislation, policies, actions or omissions of the Crown that are alleged to breach the promises made in the Treaty of Waitangi.
